# Changes in the TCRβ Repertoire and Tumor Immune Signature From a Cutaneous Melanoma Patient Immunized With the CSF-470 Vaccine: A Case Report

**DOI:** 10.3389/fimmu.2018.00955

**Published:** 2018-05-03

**Authors:** Mariana Aris, Alicia Inés Bravo, María Betina Pampena, Paula Alejandra Blanco, Ibel Carri, Daniel Koile, Patricio Yankilevich, Estrella Mariel Levy, María Marcela Barrio, José Mordoh

**Affiliations:** ^1^Centro de Investigaciones Oncológicas-Fundación Cáncer, Buenos Aires, Argentina; ^2^Unidad de Inmunopatología, Hospital Interzonal General de Agudos Eva Perón, San Martín, Argentina; ^3^Instituto de Investigaciones Biotecnológicas (IIB-INTECH) - CONICET, Universidad Nacional de San Martín (UNSAM), Buenos Aires, Argentina; ^4^Instituto de Investigación en Biomedicina de Buenos Aires (IBioBA) - CONICET, Buenos Aires, Argentina; ^5^Instituto Médico Especializado Alexander Fleming, Buenos Aires, Argentina; ^6^Fundación Instituto Leloir, Instituto de Investigaciones Bioquímicas de Buenos Aires (IIBBA) - CONICET, Buenos Aires, Argentina

**Keywords:** cutaneous melanoma, CSF-470 vaccine, T-cell receptor β immune repertoire, cancer immunogram, tumor immune infiltration

## Abstract

The allogeneic therapeutic vaccine CSF-470 has demonstrated a significant benefit over medium-dose IFNα2b in the distant metastasis-free survival for stages IIB–IIC–III cutaneous melanoma patients in a randomized phase II/III clinical trial (CASVAC-0401, NCT01729663). At the end of the 2-year CSF-470 immunization protocol, patient #006 developed several lung and one subcutaneous melanoma metastases; this later was excised. In this report, we analyzed the changes throughout vaccination of immune populations in blood and in the tumor tissue, with special focus on the T-cell repertoire. Immunohistochemistry revealed a marked increase in CD8^+^, CD4^+^, and CD20^+^ lymphocytes infiltrating the metastasis relative to the primary tumor. Lymphocytes were firmly attached to dying-tumor cells containing Granzyme-B granules. Whole-exon sequencing assessment indicated a moderate-to-high tumor mutational burden, with BRAF^V600E^ as the main oncogenic driver. Mutational signature presented large numbers of mutations at dipyrimidines, typical of melanoma. Relevant tumor and immune-related genes from the subcutaneous metastasis were addressed by RNA-Seq analysis, revealing expression of typical melanoma antigens and proliferative tumor-related genes. Stimulatory and inhibitory immune transcripts were detected as well as evidence of active T-cell effector function. Peripheral blood monitoring revealed an increase in CD4^+^ and CD8^+^ cells by the end of the immunization protocol. By CDR3-T-cell receptor β (TCRβ) sequencing, generation of new clones and an increase in oligoclonality was observed in the peripheral T-cells immune repertoire throughout immunization. A shift, with the expansion of selected preexisting and newly arising clones with reduction of others, was detected in blood. In tumor-infiltrating lymphocytes, prevalent clones (50%) were both new and preexisting that were expanded in blood following CSF-470 immunization. These clones persisted in time, since 2 years after completing the immunization, 51% of the clones present in the metastasis were still detected in blood. This is the first report of the modulation of the TCRβ repertoire from a melanoma patient immunized with the CSF-470 vaccine. After immunization, the changes observed in peripheral immune populations as well as in the tumor compartment suggest that the vaccine can induce an antitumor adaptive immune repertoire that can reach tumor lesions and persists in blood for at least 2 years.

## Introduction

Our group has developed the therapeutic vaccine CSF-470, an allogeneic mixture of four-lethally irradiated cutaneous melanoma (CM) cell lines co-adjuvated with bacillus Calmette–Guerin (BCG) and recombinant human granulocyte macrophage colony-stimulating factor (rhGM-CSF), for the adjuvant treatment of stages IIB–IIC–III CM patients. Recently, in a randomized phase II study, CSF-470 vaccine has demonstrated a significant benefit in distant metastasis-free survival versus interferon alpha 2b (IFNα2b), with good tolerability and evidence of induction of adaptive and innate immune responses (1). Assessment of the mechanisms leading to clinical benefit in adjuvancy is challenging, and monitoring different aspects of the tumor–immune system axis can be of vital importance. Along with this line, a cancer immunogram recently proposed by Blank et al. aims to analyze multifactorial aspects related to tumor immunotherapy, with focus on T cell function, including tumor foreignness, general immune status, immune cell infiltration, presence of immune checkpoints (ICKs) and other inhibitory molecules, and sensitivity to immune effectors (2). Furthermore, integral exomic and transcriptomic analysis of non-microdissected metastatic melanoma tissues allows determining tumor mutations and expression of genes related to tumor biology and immune status, which can have an impact on patient’s outcome. Recently, the usefulness of targeted RNA-Seq in profiling a panel of immune transcripts has been proposed to measure the abundance of immune transcripts in tumor biopsies to develop a gene-expression profile instrument to predict clinical response to immunotherapy (3).

In this work, we present the analysis of samples obtained from a CM patient treated with the CSF-470 vaccine (CASVAC-0401 study) who developed metastases at the end of the 2-year immunization treatment, among them a subcutaneous metastasis (SC mts) which was resected. This case allowed us to study the local immune response and its relationship with changes in the peripheral blood immune response during 4 years of follow-up (F-UP). We characterized tumor-driver genes and immune profile oriented gene-expression pattern in the SC mts; dynamics of the blood immune response throughout the vaccination protocol was also followed by analysis of the T-cell receptor β (TCRβ) immune repertoire.

## Case Report

### Patient’s #006 Case Presentation and Treatment

**Figure 1:**
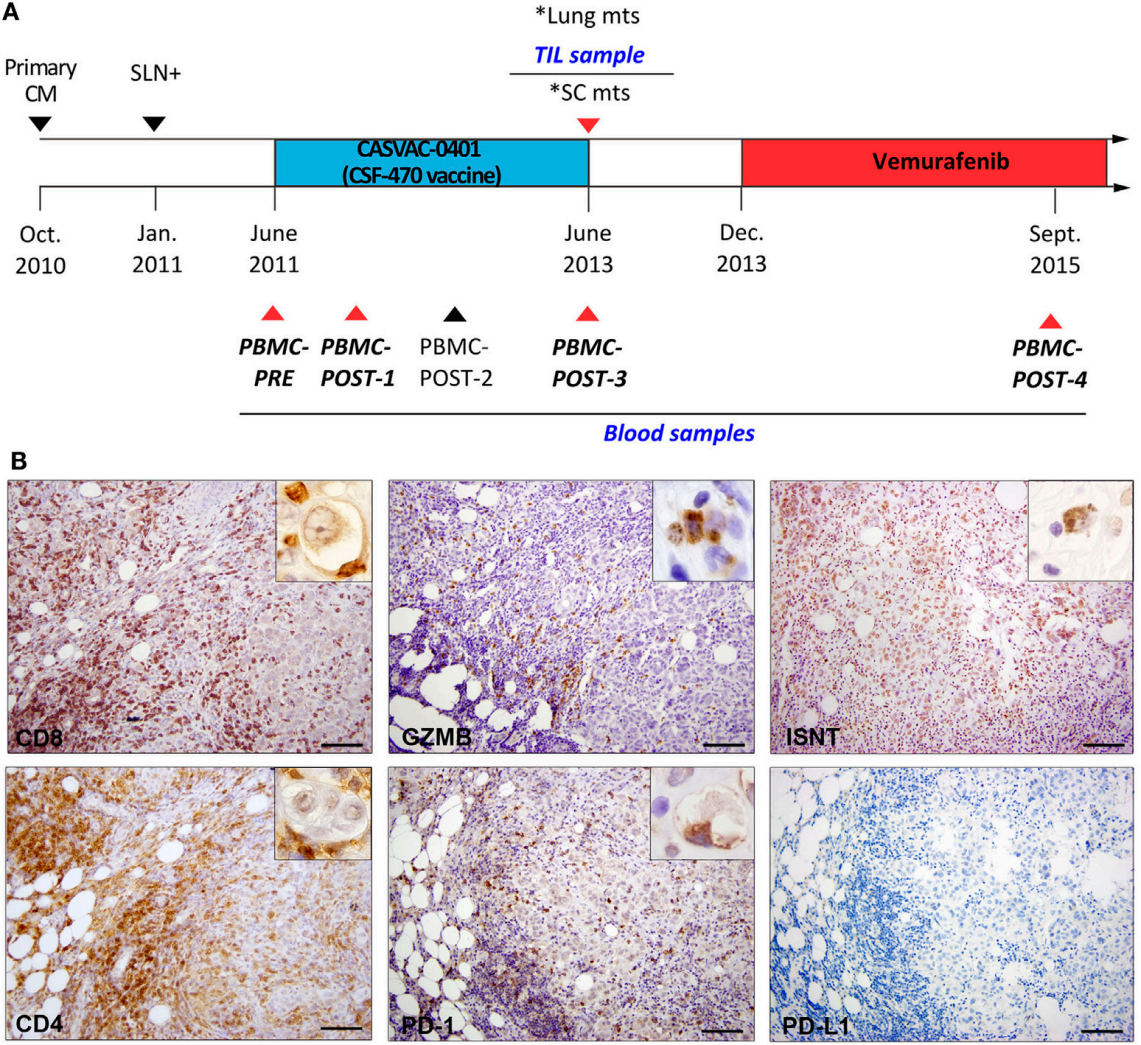
Patient #006 timeline and immune infiltrate analysis in post-vaccination subcutaneous metastasis (SC mts). **(A)** The patient was randomized to the CSF-470 vaccine arm of CASVAC-0401 study. Tumor resections of primary tumor, sentinel node, and SC mts are indicated by triangles. Peripheral blood mononuclear cell (PBMC) samples were obtained at 0 (PBMC-PRE), 6 (PBMC-POST-1), 12 (PBMC-POST-2), 24 (PBMC-POST-3), and 48 months (PBMC-POST-4) from vaccine protocol. *Lung and SC mts were detected at the same time; tumor-infiltrating lymphocytes (TIL) were obtained from the SC mts. Samples selected for T-cell receptor β sequencing are indicated with red triangles and in italics. **(B)** Representative pictures of the same region in the SC mts after IHC staining for CD8, Granzyme-B (GZMB), *in situ* nick translation (ISNT), CD4, PD-1, and PD-L1 are shown (DAB, brown). Insets show positive staining under high magnification (1,000×). Scale bars = 100 µm.

In October 2010, patient #006, a 50-year-old Caucasian woman, had a primary CM resected from her back. Three months later, an axillary sentinel lymph node with micrometastasis was excised (January 2011) (Figure 1A). After a radical axillary lymph node resection, 0/18 metastatic lymph nodes were found. In June 2011, the patient signed informed consent and entered the CASVAC-0401 study, being assigned to the CSF-470 arm. CASVAC-0401 is a single institution, randomized, open-label phase II/III study, to investigate in CM pts stages IIB, IIC, and III post-surgery (adjuvancy) the efficacy and safety (primary objectives) as well as the quality of life and immune response (secondary objectives) of the CSF-470 vaccine versus medium-dose IFN-α2b treatment (ClinicalTrials.gov: NCT01729663). Vaccine CSF-470 is an allogeneic vaccine consisting of irradiated CM cell lines plus BCG and rhGM-CSF; immunization protocol was followed as described (1). Two years later, at the end of the 13-vaccine immunization program, the patient developed SC and lung metastases; the SC mts was resected (June 2013); a part of the biopsy was formalin-fixed and paraffin-embedded for histological analyses, and a part was preserved at −80°C in RNAlater (Ambion). Bilateral lung nodules of up to 1-cm diameter were detected by a CT scan. Since this tumor had the BRAFV600E mutation, the patient started treatment with Vemurafenib 960 mg bid on December 2013, achieving a complete response within 3 months of treatment. This patient remained stable up to December 2016, when she developed lung, bone, and brain mts for which she received holocraneal radiotherapy. The last contact was on June 2017, and the patient was still alive.

### Immune Assessment of Tumor Tissues

The primary tumor in the back was a nodular, epithelioid CM with 3.0 mm Breslow thickness, micro-ulceration, vessel infiltration by tumor cells, and 15% Ki-67^+^ tumor proliferating cells (Figure S1I in Supplementary Material). The SC mts that developed in the patient’s back, in the vicinity of the previous scar, had defined expansive edges, with areas of epithelioid tumor cells adjoined by zones of dense fibrosis and zones fulfilled with brisk lymphocyte infiltration (Figure S1II in Supplementary Material). A decreasing proliferative index (PI) from 40% Ki-67^+^ tumor cells in areas without lymphocytic infiltration, to 15% Ki-67^+^ in highly infiltrated zones was observed. Ki-67^+^ lymphocytes were also distinguished (not shown). In the areas of brisk lymphocyte infiltration, necrotic/apoptotic tumor cells, distinguished by cell shrinkage with nuclear disruption, were observed surrounded by immune cells, mainly CD8^+^ and CD4^+^, some of them firmly attached (Figure 1B); Granzyme-B staining revealed positive cytosolic granules in tumor cells. *In situ* nick translation (ISNT) detected positive nuclei and vesicles within dying-tumor cells, confirming the presence of apoptotic tumor cells in close contact with lymphocytes (Figure 1B). Few PD-1^+^ lymphocytes were observed attached to tumor cells, PD-L1 expression was negative (Figure 1B). Also, CD45Ro^+^ and CD68^+^ cells were seen in the same areas (data not shown).

**Figure 2:**
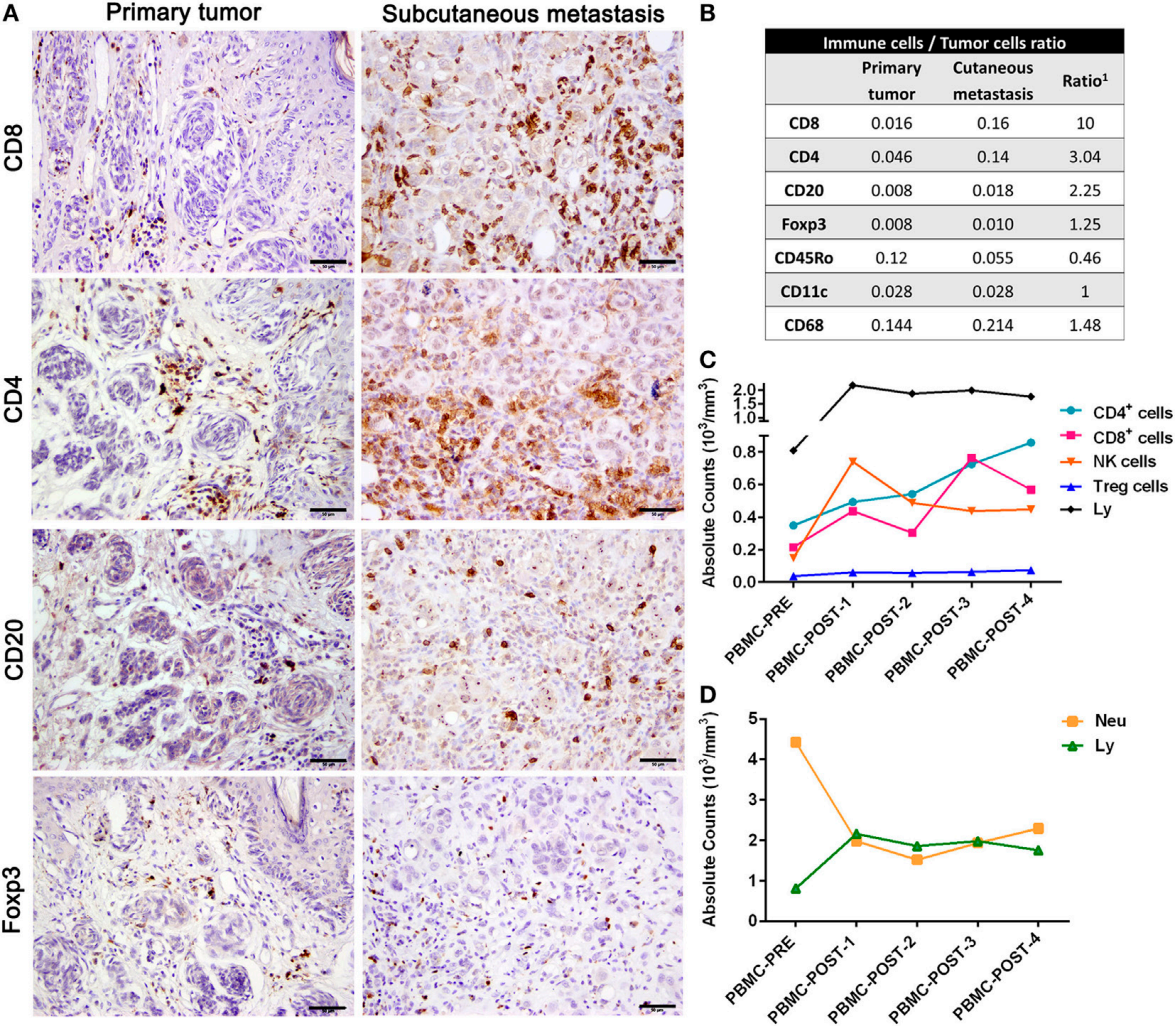
Immune profiling of patient #006. **(A)** Representative pictures of the primary tumor and subcutaneous metastasis biopsies infiltrated by CD8^+^, CD4^+^, CD20^+^, and Foxp3 lymphocytes determined by IHC (DAB, brown). **(B)** After counting total biopsy area, immune-to-tumor cell ratios were determined for each tumor biopsy as well as the relative ratio between primary tumor and SC metastasis (^1^). **(C)** Absolute counts of total lymphocytes, T CD4^+^, T CD8^+^, Treg, and NK cells from PBMC were determined throughout CASVAC-0401 treatment and follow-up by flow cytometry; also, absolute counts of lymphocytes (Ly) and neutrophils (Neu) are shown **(D)**. Scale bars = 50 µm.

Comparative analysis of the immune cells infiltrating the primary tumor and the SC mts revealed an increase mainly of CD8^+^, CD4^+^, and CD20^+^ lymphocytes (Figure 2A). Total cell counts across whole tumor sections were performed to determine immune-to-tumor cell ratio; CD8^+^, CD4^+^, and CD20^+^ lymphocytes increased 10-fold, 3-fold, and 2-fold, respectively, in the SC mts with respect to the primary tumor, while Foxp3^+^ cells were scarce (Figure 2B). CD11c^+^ and CD68^+^ antigen (Ag)-presenting cells were also frequently detected; CD45Ro^+^ memory cells decreased at the SC mts (Figure 2B). NKp46^+^ cells (NK cells) were scarce and distributed in the periphery of the SC mts (data not shown).

### Immune Assessment of PBMC Samples

Peripheral blood lymphocyte analyses revealed a sustained increase in total lymphocytes, and of T CD4^+^, T CD8^+^, and NK cells during CSF-470 treatment (Figure 2C). Whereas the marked increase in NK cells was already detected 6 months following the first immunization, CD4^+^ and CD8^+^ T cells increased by the end of the protocol (24 months); low and stable levels of Tregs were observed throughout treatment (Figure 2C). Two years after completing the immunization protocol, CD4^+^ increased while CD8^+^ levels decreased, although remaining at higher levels than at baseline; NK and Tregs stayed still (Figure 2C). Also, an increase in serum immunoglobulin G Abs recognizing both CSF-470 cells as well as patient’s autologous tumor cells was reported elsewhere (1). Neutrophils/lymphocytes ratio sharply diminished during CSF-470 immunization (Figure 2D).

### Tumor Mutational Burden and Gene-Expression Analysis of SC mts

Patient #006 SC mts was highly mutated. After elimination of germline variants, somatic mutations revealed a total number of 712 SNPs and 33 InDels. We found 239 mutations in coding sequences: 7 were insertions; 72 were synonymous variants, and 156 were missense mutations. Tumor mutational burden was moderate to high (TMB = 745 variants/50.39 Mb = 14.8), suggesting that immunogenic neoantigens might have been generated. Among driver genes, BRAF^V600E^, FBXW7^S608T^, and CIC^Δ374^ heterozygous mutations were found. Analysis of mutational signature revealed fingerprints related to failure of DNA double-strand break-repair by homologous recombination; large numbers of CC>TT dinucleotide mutations at dipyrimidines due to ultraviolet light exposure, typical of melanoma, were also found (Figure S2 in Supplementary Material).

RNA-Seq of the SC mts revealed the expression of several melanoma-associated Ags, many of them shared with the CSF-470 vaccine cells (Table 1) (1). Also, typical melanoma markers, such as MC1R, vimentin, MITF, CD63, and LADH were abundant (Table 1). Since the whole tissue biopsy was sequenced, containing both tumor and brisk immune cells infiltration, we focused on transcripts that could reflect the immune expression profile of the tumor microenvironment. Human leucocyte antigen (HLA) class I (HLA-A) and β2M transcripts were abundantly expressed (Table 1). Stimulatory ICKs such as CD40, as well as inhibitory ICKs such as CTLA4, B7-H3, TIGIT, Tim-3, and LAG-3 were detected. PD-1/PD-L1 transcripts were scarce. Immunosuppressive molecules IDO, galectins 1, 3, and 9, and HLA-E were expressed, but no IL-10 was detected. Markers of inflammation such as TNF-α, CSF1R, and CXCL8 were also abundant. We also searched in our RNA-Seq results for a panel of transcripts related to T-cell receptor signaling, tumor infiltration by immune cells, and other immunological functions that are key for anticancer immunosurveillance (3). We found elevated expression of leukocyte population markers; Ag processing and presenting genes; and IFN-I and II response genes. Finally, transcripts of CD4^+^ and CD8^+^ T cells markers, TCR co-expression and receptor signaling, and granzyme and perforin genes were abundantly detected (Table S1 in Supplementary Material).

**Table 1:** Abundance of selected immune-related transcripts from subcutaneous metastasis from patient #006.

Associated gene name	FPKM	Associated gene name	FPKM	Associated gene name	FPKM
**Melanoma markers**		**TCR co-expression and signaling**		**Human leucocyte antigen (HLA) expression**	
CD63	3,487.90	CD3D	51.37	HLA-A	890.74
Vimentine	2,928.7	CD8B	14.82	HLA-DRB4	195.09
CTSD	408.69	CD3E	11.16	HLA-B	118.11
CDK4	268.04	CD8A	7.47	HLA-DRA	94.56
LDHA	227.53	LCK	7.40	HLA-DRB3	81.38
SPARC	189.08	CD3G	1.71	HLA-DRB1	34.71
MITF	20.21	NFKBIA	31.67	HLA-C	2.55
MC1R	9.82	PTPN6	10.73		
VEGFA	3.38	ZAP70	6.67		
		CBLB	1.55		

**Melanoma differentiation antigens**		**Cytokines and chemokines**		**Immune checkpoints and ligands**	

PMEL/gp100	528.89	CXCL1	24.48	PD-1	1.84
MLANA/MART-1	289.90	CXCL10	23.69	CD274/PD-L1 (PD-1)	0.81
TYR	63.37	CXCL9	16.14	PDL2 (PD-1)	1.74
RAB38 (NY-MEL-1)	33.46	CSF1R	13.12	CTLA4	1.82
DCT/TRP2	30.33	CXCL8	4.68	CD80 (CTLA4)	0.57
		TNF	1.51	CD86 (CTLA4)	5.95
		IFNGR2	65.96	TIM-3	6.70
		IFNGR1	19.59	LSGAL9 (TIM-3)	37.83
				CD40	12.74
				CD40LG (CD40)	0.29
				ICOS	0.91
				ICOSLG (ICOS)	1.48

**Cancer testis antigens**		**Immunosuppressive molecules**		**Cytolytic activity**	

MAGED2	289.16	LGALS1	4,643.39	GZMA	26.61
GPR143	125.40	LGALS3	2,165.00	GZMK	24.73
MAGED1	71.97	HLA-E	536.57	GZMB	14.79
IL13RA2	2.72	TGFB1	21.45	GNLY	14.35
SPAG9	1.98	IDO1	13.05	GZMH	11.50
		IL-10	0.39	PRF1	4.35

*Abundance of selected transcripts is expressed in fragments per kilobase of exon per million reads mapped (FPKM)*.

### Diversity of the TCRβ Immune Repertoire in Blood and SC mts

An increase in CD4^+^ and CD8^+^ lymphocytes in the peripheral compartment after immunization with the CSF-470 vaccine, as well as a brisk infiltration of CD4^+^ and CD8^+^ lymphocytes in the SC mts, was observed (Figure 2A). We analyzed the dynamics of the T-cell repertoire throughout immunization with the CSF-470 vaccine, since a central question was whether TIL clones were already present in blood previous to CSF-470 immunization, which expanded and were recruited to the SC mts; or if new T-cell clones arose after immunization. We performed NGS of the CDR3 region of the TCRβ repertoire genes, since within the TCR, this region contains the highest variability, making it a suitable molecular identifier for tracking individual T-cell clones (4). TCRβ sequence analyses were performed in the SC mts TIL and in PBMC samples obtained before (PBMC-PRE), during CSF-470 immunization (PBMC-POST-1 and PBMC-POST-3), and 2 years after completing the vaccine protocol (PBMC-POST-4) (Figure 1A; Tables S2–S6 in Supplementary Material).

**Figure 3:**
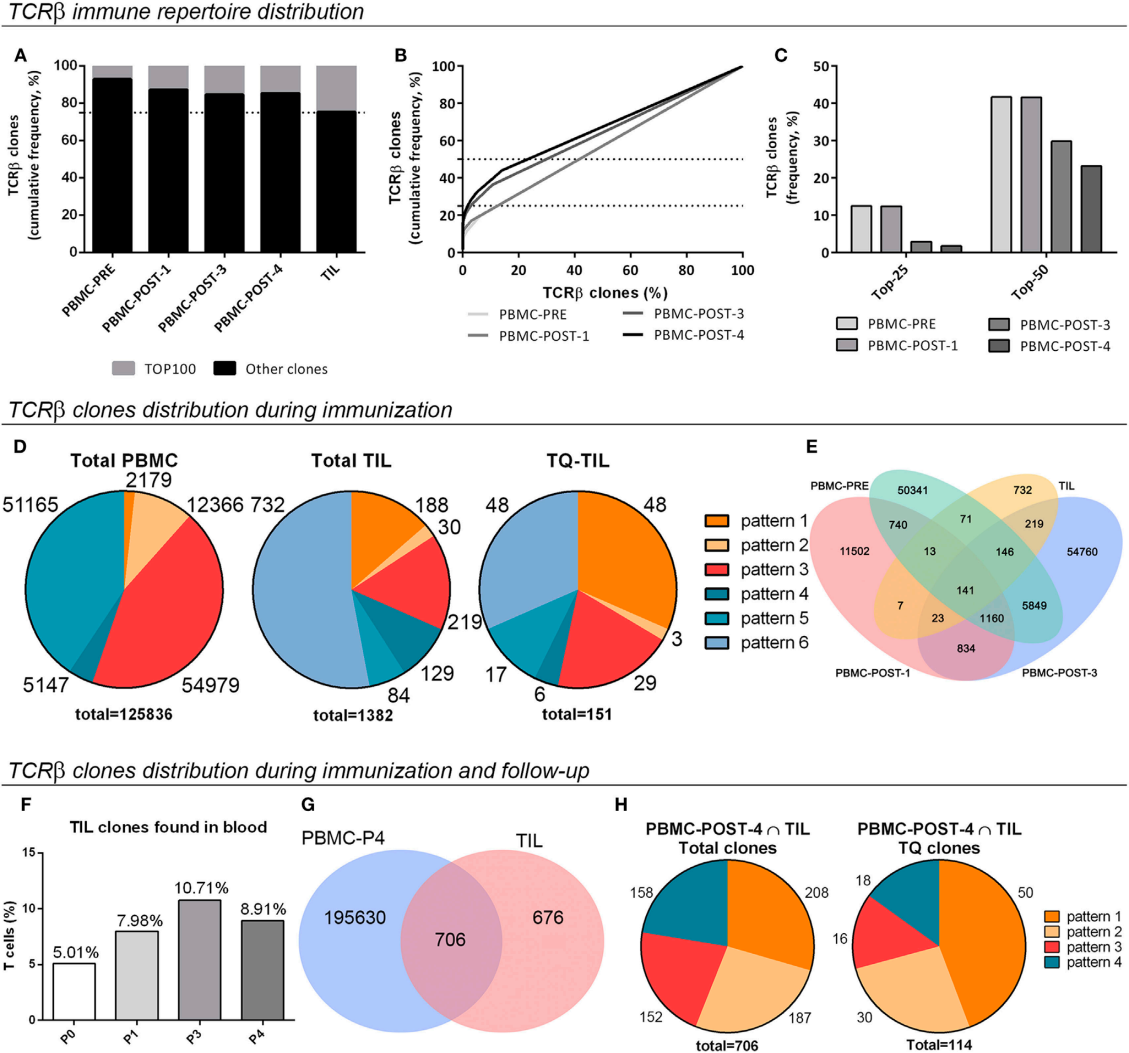
Distribution and tracking of the T-cell receptor β (TCRβ) immune repertoire in peripheral blood mononuclear cells (PBMC) and subcutaneous metastasis from patient #006. **(A)** Percentage of total TCRβ clones covered by the TOP100 clones, the 100 most frequent ones (nucleotide sequences). **(B)** Total TCRβ clones (%), from highest to lowest, in function of their cumulative frequency (nucleotide sequences). **(C)** The proportion of TCRβ clones (%) that constitute the TOP-25 or TQ clones, and the TOP-50 clones are shown (TOP-25/50, the proportion of clones that make up the top cumulative frequency of 25/50). **(D)** Pattern distribution of TCRβ clones in total PBMC, total tumor-infiltrating lymphocytes (TIL), and Top-Quartile TIL (TQ-TIL); the number of clones for each pattern and total clones analyzed in each case is indicated. Patterns: 1, present at baseline, increases in time; 2, absent at baseline, increases in time; 3, absent at baseline, detected at POST-3 sample; 4, present at baseline, decreases in time; 5, present at baseline, absent in time; and 6, absent at blood, present in tumor. **(E)** Common sequences among PBMC-PRE, PBMC-P1, PBMC-P3, and TIL samples. **(F)** The proportion of TIL clones present in blood at different times. **(G)** Venn diagram illustrating common clones among TIL and POST-4 samples regarding amino acid sequences. **(H)** Distribution in tracking patterns of total and TQ-TIL clones shared with PBMC POST-4. Pattern 3: absent at baseline, detected at POST-4 sample.

To describe the diversity of the TCRβ immune repertoire of each sample, considering the number of unique receptors (richness) and their relative abundances (evenness), different analyses were performed. Analyses of the 100 most frequent TCRβ clones (TOP100) indicated an increment in their cumulative frequency throughout immunization, supporting preferential expansion of a subpopulation of T-cell clones (Figure 3A). TOP100 TIL clones from the SC mts showed an oligoclonal TCRβ repertoire almost attaining the top-quartile most frequent clones (TQ) (Figure 3A). Also, the frequency of each TCRβ clone in the immune repertoire (%), ordered from highest to lowest, was plotted in function of its cumulative frequency (%) to compare blood samples (Figure 3B). PBMC-PRE sample presented a linear pattern, which is related to an homogeneous distribution of the TCRβ repertoire. A similar pattern was observed for PBMC-POST-1. Instead, a sigmoideal pattern was observed for PBMC-POST-3 sample, as less T-cell clones make up more of the entire repertoire. Thus, there was a progressive enrichment in a subset of T-cell clones. A similar pattern was observed for PBMC-POST-4 (Figure 3B). When analyzing the proportion of clones that make up the top cumulative frequency of 25 [TOP-25 or Top-Quartile (TQ)] or 50 (TOP-50), it decreases over time; further supporting the expansion of a subpopulation of T-cell clones (Figure 3C).

### Dynamics of the TCRβ Immune Repertoire Throughout Immunization With the CSF-470 Vaccine

To further analyze the changes induced in the TCRβ immune repertoire of patient #006 throughout immunization with the CSF-470 vaccine, six different patterns for tracking T-cell clones in blood were defined (Figure 3D; Figure S3A in Supplementary Material). A shift in the TCRβ repertoire composition in blood was observed, with a significant proportion of pre-existing clones increasing its frequency (pattern 1) or newly arising throughout immunization at 12 and 48 months (pattern 2 and pattern 3). Opposite to that, a proportion of clones either decreased (pattern 4) or became undetectable by the end of the immunization protocol (pattern 5).

### Characterization of the TCRβ Immune Repertoire Infiltrating the SC mts

Analysis of shared amino acid sequences among TIL and blood samples revealed that all samples shared 10% of the clones, and 16% were shared by TIL and PBMC-POST3 (Figure 3E). When total TIL clones were analyzed considering the tracking patterns for peripheral clones, 53% of TIL clones were only detected at the tumor microenvironment (pattern 6); 13.6% corresponded to preexisting clones that expanded during immunization (pattern 1), clones emerging during immunization and detected at 12 months (2.2%, pattern 2) and 24 months by the end of immunization (15.9%, pattern 3) (Figure 3D). 9.3 and 6.0% of TIL clones diminished their frequency in blood or were no longer detected. When looking at the most frequent Top-Quartile TIL clones (TQ-TIL), preexisting clones (pre-vaccination) were enriched (pattern 1, 31.6%), prevailing along with late-emerging clones (pattern 3, 19.8%) (Figure 3D). 58% of TQ-TIL clones corresponded to pattern 1 clones that have expanded in blood at least three times; and 45% of TQ-TIL clones were in the 2.4% most abundant clones in PBMC-POST-3 sample (pattern 3) (data not shown). This supports the idea that a considerable proportion of abundant TIL clones (TQ-TIL) are among those that were expanded in blood, either preexistent or emerging post-vaccination. TQ-TIL clones detected only at the tumor microenvironment accounted for 31.6% (pattern 6) (Figure 3D).

A F-UP blood sample, obtained 2 years after patient #006 completed the protocol and after 2 years of intermittent treatment with vemurafenib (PBMC-POST-4 sample), revealed that TCRβ clones found in the tumor microenvironment could still be detected in circulation. Indeed, the cumulative frequency of TIL clones detected in blood doubled from 5% (PBMC-PRE) to 10.7% total frequency (PBMC-POST-3) (Figure 3F). Two years later (PBMC-POST-4 sample), TIL clones were still detected in blood with a cumulative frequency of 8.9% (Figure 3F). Actually, 51% of total TIL clones were shared with the POST-4 sample (Figure 3G). These 706 common clones were distributed mainly in four patterns, 56% of them corresponded to patterns 1 and 2; while 21.6% were detected only at POST-4 blood sample (pattern 3) (Figure 3H; Figure S3 in Supplementary Material). When looking at TQ-TIL clones shared with PBMC-POST-4, expanded preexisting and clones arising during immunization accounted for 70.8% (patterns 1 and 2) (Figure 3H). These results suggest that CSF-470 vaccination in patient #006 modulated the repertoire of T-cell clones and their frequency, and that particularly those clones expanded during immunization persisted at least 24 months after the end of the vaccination protocol.

T cells with shared Ag specificities should share conserved CDR3 structural domains and amino acid sequences, as they recognize the same epitopes (5). An oligoclonal pattern of Vβ expression should thus be observed if Ag-driven stimulation causes clonal expansion. We found that this patient presented a Vβ gene usage distribution that was mainly conserved in time in peripheral PBMC and TIL (Figure S4A in Supplementary Material). When analyzing total TIL, as well as TIL that persisted in time (PBMC-POST-4 ∩ TIL) according to their distribution patterns, the Vβ gene usage distribution was similar (Figures S4B,C in Supplementary Material).

## Discussion

We present here the analysis of samples from patient #006 enrolled in the CASVAC-0401 trial treated with the CSF-470 vaccine, who simultaneously developed one SC and several lung mts after receiving 13 immunizations during 2 years (1). The SC mts was excised and offered the opportunity to correlate the tumor and peripheral immune responses during immunization and 2-year F-UP. Immunohistochemical analysis of the SC mts revealed a 10-fold increase of CD8^+^ lymphocytes with respect to the primary tumor; less so (3-fold) for CD4^+^ cells. Evidence suggesting that CD8^+^, CD4^+^, and macrophages effector cells were actually killing tumor cells was obtained, supported by ISNT and GZMB expression. The reason why some tumor areas are infiltrated whereas other parts of the tumor appear unreached is not known. It does not appear to be due to an unequal distribution of immune suppressor cells, since FOXP3^+^ Tregs were scarce and even macrophages appeared to be lysing tumor cells.

To elucidate if immune effector cells preexisted before vaccination and/or were triggered by it, we compared the TCRβ immune repertoire of blood and TIL. Peripheral blood monitoring revealed an increase in CD4^+^ and CD8^+^ cells by the end of the immunization protocol. Along vaccination, a shift in the peripheral CDR3-TCRβ immune repertoire, with the expansion of preexisting clones (2% of total clones), the appearance of new clones (50% of total), and diminution of others (45% of total), was observed. Although preexisting TCRβ clones might recognize private or melanoma-associated Ags previous to vaccination, their expansion suggests a positive effect of vaccination. A central question, however, was if vaccination induced lymphocyte clones able to travel into the metastatic foci. Most important, analysis of TIL clones revealed that 29.5% of them were preexisting or late-emerging in blood. Therefore, vaccination induced the generation and expansion of T cell clones that were able to migrate into tumor sites. When looking at the most frequent clones (TQ-TIL), which might be the more relevant for antitumor function (6), preexisting as well as late-emerging clones became prevalent, accounting for 51% of the total. The proportion of TIL clones present in the blood repertoire doubled in time throughout immunization, remaining at levels higher than detected at baseline. An F-UP blood sample, obtained 2 years after completing the immunization protocol, showed that half of TIL clones could still be detected; although it should be pointed that patient #006 was treated with vemurafenib for 21 months when the F-UP sample was taken (09/2015). The patient achieved a complete response to vemurafenib of 31 months duration, after which she progressed with lung, bone, and brain mts. There is evidence that vemurafenib does not interfere with the viability or functionality of T cells (7, 8). In this case, we could still detect TCRβ TIL clones 2 years after completing immunization with the CSF-470 vaccine; conceivably, further Ag-input was delivered to the immune system due to tumor cell lysis as suggested by the clinical complete response to vemurafenib. The correlate of the findings in the SC mts with the lung mts could not be performed to determine if a similar immune reaction was taking place. It is nevertheless remarkable that the patient achieved a complete response with vemurafenib for more than 2 years, although the relationship between this response and the immune reaction may not be ascertained.

In line with our findings, Valmori et al. reported a functional and structural longitudinal analysis of the TCR of circulating CD8 T cells specific for the HLA-A2-restricted immunodominant epitope from the melanocyte differentiation Ag Melan-A in a melanoma patient who developed a vigorous and sustained Ag-specific T cell response following vaccination with the corresponding synthetic peptide. Some of these T cell clones were also identified at a metastatic tumor site, concluding that vaccine stimulation leads to the selection of high-avidity T cell clones of increased tumor reactivity that independently evolve within oligoclonal populations (9). Also, Carreno et al. reported that immunization with a dendritic cell-based personalized vaccine targeting neoantigens and gp100 peptides in three melanoma patients, promoted a diverse neoantigen-specific T cell receptor repertoire in terms of both TCRVβ usage and clonal composition. In pre-vaccination CD8^+^ T cell populations, as few as one and as many as 10 unique TCRβ clonotypes per neoantigen were identified. Vaccination increased the frequency of most existing pre-vaccine TCRβ clonotypes and revealed new clonotypes for all neoantigens tested. For both dominant and subdominant neoantigens, the TCRβ repertoire was increased significantly after vaccination (10).

Integral analysis of a non-microdissected metastatic melanoma tissue allowed us to carry out an exploratory search for expression of genes related to tumor biology and to the presence of immune populations infiltrating the tumor. Immunohistochemistry and RNA-Seq results suggest that immunization with CSF-470 vaccine has triggered T-cell priming, as seen by brisk tumor immune infiltration. Other inhibitory immune pathways might be activated, as suggested by the abundance of B7-H3 and galectin 1 (11), galectin 3 (12), and galectin 9 transcripts (13, 14). Also, Tim-3 and LAG-3 transcripts were detected; expression of these exhaustion molecules was previously reported in tumor-reactive infiltrating lymphocytes following PD-1 immune checkpoint blockade (ICKB) (15). Only a low proportion of TIL expressed PD-1, probably preexisting Ag-experienced T cells, some of them in contact with tumor dying cells. Low levels of PD-1^+^ expression found in TIL can be due to the fact that PD-1 is transiently expressed in T cells activation by Ags (16). Since high PD-1 expression levels are achieved in chronic microenvironments, probably this inflamed tumor was removed before the establishment of local immune escape mechanisms driven by chronic tumor Ag exposure (17). Also, high levels of LADH were detected, indicative of a highly proliferative tumor, supported by Ki-67^+^ PI and activation of MAPK pathway through BRAF mutation. Along with this line, a recent work on a cohort of metastatic CM patients reported that response to PD-1 ICKB was better predicted when integrating T cell invigoration to tumor burden. Many TCRβ clones detected in pre-treatment TIL were also detected in blood only after treatment thus supporting the induction of an immune response. The clinical outcome was better predicted when the ratio of re-activated exhausted T cells to tumor burden was taken into account (18).

## Concluding Remarks

This is the first report of the TCRβ repertoire from a CM patient immunized with the CSF-470 vaccine. The changes observed in peripheral immune populations as well as in the tumor compartment after immunization suggest that the vaccine can induce an antitumor adaptive immune repertoire that can reach tumor lesions and persist in blood for at least 2 years.

## Ethics Statement

The CASVAC-0401 study was carried out in accordance with the recommendations of the “Ethics Committee of the Instituto Médico Especializado Alexander Fleming” with written informed consent from all subjects. All subjects gave written informed consent in accordance with the Declaration of Helsinki. The informed Consent included the authorization to publish the results obtained, providing anonymity was assured the protocol was approved by the “Prof. Luis María Zieher Independent Ethics Committee for Clinical assays in Clinical Pharmacology” (Argentina) and “Ethics Committee of the Instituto Médico Especializado Alexander Fleming (Buenos Aires, Argentina), and by the local Regulatory Agency (ANMAT—Argentina) (Disposition 1299/09).” The “Ethics Committee of the Instituto Médico Especializado Alexander Fleming (Buenos Aires, Argentina)” is reputed by the Central Ethics Committee of the City of Buenos Aires (Argentina).

## Author Contributions

MA: collection and assembly of data; data analysis and interpretation; and manuscript writing. AB, MP, and PB: collection and assembly of data; data analysis and interpretation. IC and DK: data analysis and interpretation. PY, EL, and MB: data analysis and interpretation; manuscript writing. MB is the Sub-Investigator of the CASVAC-0401 study. JM: conception and design of the study; collection and assembly of data; data analysis and interpretation; and manuscript writing. JM is the Principal Investigator of the CASVAC-0401 study.

## Conflict of Interest Statement

The authors declare that the research was conducted in the absence of any commercial or financial relationships that could be construed as a potential conflict of interest.

## Supplementary Material

Table S1 Transcripts levels in subcutaneous metastasis (SC mts) from patient #006 [fragments per kilobase of exon per million reads mapped (FPKM)].

Table S2 Peripheral blood mononuclear cells-PRE productive rearrangements.

Table S3 Peripheral blood mononuclear cells-POST-1 productive rearrangements.

Table S4 Peripheral blood mononuclear cells-POST-3 productive rearrangements.

Table S5 Peripheral blood mononuclear cells-POST-4 productive rearrangements.

Table S6 Tumor-infiltrating lymphocytes productive rearrangements.

## Abbreviations

Abs, antibodies; Ags, antigens; BCG, bacillus Calmette–Guerin; CM, cutaneous melanoma; DCs, dendritic cells; FFPE, formalin-fixed and paraffin-embedded; FPKM, fragments per kilobase of exon per million reads mapped; F-UP, follow-up; GZMB, Granzyme B; HLA, human leucocyte antigen; ICKB, immune checkpoint blockade; ICKs, immune checkpoints; Igs, immunoglobulins; pts, patients; PBMC, peripheral blood mononuclear cells; PI, proliferative index; QOL, quality of life; rhGM-CSF, recombinant human granulocyte-macrophage colony-stimulating factor.
