# Supplementary material for: Changes in the TCRβ Repertoire and Tumor Immune Signature From a Cutaneous Melanoma Patient Immunized With the CSF-470 Vaccine: A Case Report
Source: Front Immunol. 2018 May 3;9:955. doi: 10.3389/fimmu.2018.00955 (PMC5944263; doi:10.3389/fimmu.2018.00955)
Supplement: Table S1 — Transcripts levels in subcutaneous metastasis (SC mts) from patient #006 [fragments per kilobase of exon per million reads mapped (FPKM)]. [file table_1.PDF]

Symbol	SC-mts #006_FPKM	Gene function
**CD44**	**162.48**	**Adhesion,migration**
**ITGB1**	**45.83**	**Adhesion,migration**
**ADGRE5**	**31.29**	**Adhesion,migration**
**CD53**	**31.04**	**Adhesion,migration**
**CD47**	**18.93**	**Adhesion,migration**
**NECTIN2**	**16.63**	**Adhesion,migration**
**PECAM1**	**16.60**	**Adhesion,migration**
**ITGAE**	**5.51**	**Adhesion,migration**
**IGF1R**	**3.14**	**Adhesion,migration**
**CD38**	**2.09**	**Adhesion,migration**
NCAM1	0.50	Adhesion,migration
ITGA1	0.37	Adhesion,migration
CD226	0.34	Adhesion,migration
**CD83**	**10.88**	**Antigen presentation**
**CD1D**	**3.23**	**Antigen presentation**
**CD1C**	**2.79**	**Antigen presentation**
**CD74**	**1625.77**	**Antigen processing**
**HLA-A**	**890.74**	**Antigen processing**
**HLA-E**	**536.57**	**Antigen processing**
**HLA-DPA1**	**456.05**	**Antigen processing**
**HLA-F**	**144.16**	**Antigen processing**
**HLA-DMA**	**126.86**	**Antigen processing**
**HLA-B**	**118.11**	**Antigen processing**
**HLA-DRA**	**94.56**	**Antigen processing**
**HLA-DMB**	**75.29**	**Antigen processing**
**HLA-DPB1**	**36.38**	**Antigen processing**
**HLA-DRB1**	**34.71**	**Antigen processing**
**HLA-DOA**	**8.88**	**Antigen processing**
**HLA-F-AS1**	**4.68**	**Antigen processing**
**HLA-DQB2**	**3.91**	**Antigen processing**
**HLA-DOB**	**2.84**	**Antigen processing**
**HLA-C**	**2.55**	**Antigen processing**
**HLA-DQA2**	**2.48**	**Antigen processing**
HLA-DQA1	0.28	Antigen processing
HLA-G	0.20	Antigen processing
**GADD45GIP1**	**430.70**	**Apoptosis**
**TNFSF10**	**20.50**	**Apoptosis**
**BCL2L11**	**1.64**	**Apoptosis**
**BCL2**	**1.31**	**Apoptosis**
**JCHAIN**	**160.96**	**B cell marker**
**FCGR2B**	**12.59**	**B cell marker**
**FCGR1A**	**10.89**	**B cell marker**
**TNFSF13B**	**9.69**	**B cell marker**
**SKAP2**	**5.06**	**B cell marker**
**FCRLA**	**4.79**	**B cell marker**
**CD22**	**2.21**	**B cell marker**
**POU2AF1**	**2.10**	**B cell marker**
**TNFRSF17**	**1.96**	**B cell marker**
**CD19**	**1.82**	**B cell marker**

**CD79B**	**24.90**	**B cell receptor signaling**
**CD79A**	**14.85**	**B cell receptor signaling**
**FAS**	**3.75**	**B cell receptor signaling**
**TNFRSF14**	**41.96**	**Checkpoint pathway**
**CD276**	**39.73**	**Checkpoint pathway**
**CD48**	**31.07**	**Checkpoint pathway**
**TGFB1**	**21.45**	**Checkpoint pathway**
**C10orf54**	**7.22**	**Checkpoint pathway**
**HAVCR2**	**6.70**	**Checkpoint pathway**
**CD86**	**5.95**	**Checkpoint pathway**
**ADORA2A**	**3.77**	**Checkpoint pathway**
**PVR**	**3.36**	**Checkpoint pathway**
**NT5E**	**3.09**	**Checkpoint pathway**
**ENTPD1**	**2.69**	**Checkpoint pathway**
**PDCD1LG2**	**1.74**	**Checkpoint pathway**
**CD28**	**1.55**	**Checkpoint pathway**
**TNF**	**1.51**	**Checkpoint pathway**
**EOMES**	**1.50**	**Checkpoint pathway**
**ICOSLG**	**1.48**	**Checkpoint pathway**
**CEACAM1**	**1.37**	**Checkpoint pathway**
**CD69**	**1.26**	**Checkpoint pathway**
TDO2	0.93	Checkpoint pathway
ICOS	0.91	Checkpoint pathway
CD274	0.81	Checkpoint pathway
CD244	0.75	Checkpoint pathway
TNFSF4	0.69	Checkpoint pathway
CD80	0.57	Checkpoint pathway
BTLA	0.42	Checkpoint pathway
CD160	0.28	Checkpoint pathway
TNFSF14	0.19	Checkpoint pathway
IDO2	0.09	Checkpoint pathway
**NCF1**	**11.69**	**Chemokine signaling**
**CXCR3**	**3.49**	**Chemokine signaling**
**VEGFA**	**3.38**	**Chemokine signaling**
**CCL17**	**2.56**	**Chemokine signaling**
CCR6	0.68	Chemokine signaling
CCL22	0.50	Chemokine signaling
CXCL1	0.45	Chemokine signaling
CCL20	0.13	Chemokine signaling
CCR4	0.06	Chemokine signaling
CXCR2	0.04	Chemokine signaling
**STAT5A**	**14.58**	**Cytokine signaling**
**CSF1R**	**13.12**	**Cytokine signaling**
**CXCL8**	**4.68**	**Cytokine signaling**
**IFIT2**	**4.58**	**Cytokine signaling**
**TNFSF9**	**3.91**	**Cytokine signaling**
**CCR1**	**3.31**	**Cytokine signaling**
**CSF2RB**	**1.69**	**Cytokine signaling**
**HGF**	**1.43**	**Cytokine signaling**
IL2RA	0.94	Cytokine signaling

IL7	0.78	Cytokine signaling
IL6	0.65	Cytokine signaling
**IL3RA**	**14.10**	**Dendridic cell**
**NRP1**	**3.28**	**Dendridic cell**
**HERC6**	**2.39**	**Dendridic cell**
**ITGAX**	**2.26**	**Dendridic cell**
**ZBTB46**	**1.01**	**Dendridic cell**
TLR3	0.38	Dendridic cell
CLEC4C	0.26	Dendridic cell
**CD14**	**204.77**	**Dendridic cell,macrophage**
**CMKLR1**	**7.22**	**Dendridic cell,macrophage**
**MRC1**	**3.86**	**Dendridic cell,macrophage**
**CD209**	**2.24**	**Dendridic cell,macrophage**
IL23A	0.52	Dendridic cell,macrophage
**PMEL**	**528.89**	**Drug target**
**TNFRSF4**	**24.05**	**Drug target**
**CD27**	**19.44**	**Drug target**
**SLAMF7**	**14.77**	**Drug target**
**IDO1**	**13.05**	**Drug target**
**CD40**	**12.74**	**Drug target**
**STAT3**	**11.39**	**Drug target**
**TNFRSF18**	**5.04**	**Drug target**
**LAG3**	**3.38**	**Drug target**
**MS4A1**	**2.00**	**Drug target**
**PDCD1**	**1.84**	**Drug target**
**CTLA4**	**1.71**	**Drug target**
**CD70**	**1.02**	**Drug target**
KLRD1	1.00	Drug target
IL10	0.39	Drug target
TLR9	0.27	Drug target
TNFRSF9	0.20	Drug target
IL12B	0.04	Drug target
**BATF**	**25.32**	**Helper T cells**
**CD4**	**17.51**	**Helper T cells**
**STAT6**	**15.63**	**Helper T cells**
**CCR2**	**2.69**	**Helper T cells**
**STAT4**	**1.01**	**Helper T cells**
GATA3	0.58	Helper T cells
RORC	0.03	Helper T cells
**LMNA**	**417.95**	**Housekeeping**
**TUBB**	**342.03**	**Housekeeping**
**GUSB**	**44.11**	**Housekeeping**
**SDHA**	**27.74**	**Housekeeping**
**HMBS**	**18.73**	**Housekeeping**
**ABCF1**	**18.40**	**Housekeeping**
**G6PD**	**18.08**	**Housekeeping**
**TFRC**	**6.26**	**Housekeeping**
**LRP1**	**6.20**	**Housekeeping**
**TBP**	**3.57**	**Housekeeping**
**POLR2A**	**2.18**	**Housekeeping**

**MIF**	**2643.23**	**Innate immune response**
**C1QA**	**615.22**	**Innate immune response**
**C1QB**	**465.12**	**Innate immune response**
**LYZ**	**81.55**	**Innate immune response**
**AXL**	**19.32**	**Innate immune response**
**MAPK14**	**5.85**	**Innate immune response**
**IFIH1**	**2.85**	**Innate immune response**
**TLR7**	**1.41**	**Innate immune response**
LCN2	0.89	Innate immune response
**IFI6**	**138.80**	**Interferon signaling**
**IFI35**	**94.28**	**Interferon signaling**
**MX1**	**18.73**	**Interferon signaling**
**OAS2**	**4.80**	**Interferon signaling**
**IRF4**	**3.44**	**Interferon signaling**
**IFI44L**	**1.93**	**Interferon signaling**
**DDX58**	**1.85**	**Interferon signaling**
OAS3	0.87	Interferon signaling
**LST1**	**57.41**	**Leukocyte inhibition**
**LILRB1**	**1.30**	**Leukocyte inhibition**
**SELL**	**7.67**	**Leukocyte migration**
**ITGB7**	**4.61**	**Leukocyte migration**
**VCAM1**	**4.34**	**Leukocyte migration**
**ITGAL**	**2.23**	**Leukocyte migration**
**ITGAM**	**1.44**	**Leukocyte migration**
**SH2D1A**	**1.46**	**Lymphocyte activation**
**IKZF1**	**2.01**	**Lymphocyte development**
IKZF4	0.34	Lymphocyte development
IKZF2	0.02	Lymphocyte development
**CD63**	**3487.90**	**Lymphocyte infiltrate**
**TYROBP**	**307.23**	**Lymphocyte infiltrate**
**CD52**	**165.53**	**Lymphocyte infiltrate**
**FCER1G**	**152.63**	**Lymphocyte infiltrate**
**CCL5**	**150.71**	**Lymphocyte infiltrate**
**LAPTM5**	**102.10**	**Lymphocyte infiltrate**
**CORO1A**	**87.81**	**Lymphocyte infiltrate**
**NKG7**	**58.42**	**Lymphocyte infiltrate**
**CCL21**	**46.43**	**Lymphocyte infiltrate**
**CCL2**	**43.26**	**Lymphocyte infiltrate**
**LAMP1**	**38.72**	**Lymphocyte infiltrate**
**SRGN**	**32.07**	**Lymphocyte infiltrate**
**GZMA**	**26.61**	**Lymphocyte infiltrate**
**ITGB2**	**24.88**	**Lymphocyte infiltrate**
**GZMK**	**24.73**	**Lymphocyte infiltrate**
**IL2RG**	**22.74**	**Lymphocyte infiltrate**
**CXCR4**	**20.99**	**Lymphocyte infiltrate**
**JAML**	**19.90**	**Lymphocyte infiltrate**
**CCL4**	**18.45**	**Lymphocyte infiltrate**
**CD2**	**16.85**	**Lymphocyte infiltrate**
**CTSS**	**16.61**	**Lymphocyte infiltrate**
**CD37**	**16.23**	**Lymphocyte infiltrate**

**GZMB**	**14.79**	**Lymphocyte infiltrate**
**CCL3**	**13.24**	**Lymphocyte infiltrate**
**SLAMF8**	**12.37**	**Lymphocyte infiltrate**
**GZMH**	**11.50**	**Lymphocyte infiltrate**
**IGSF6**	**9.77**	**Lymphocyte infiltrate**
**CCL18**	**7.58**	**Lymphocyte infiltrate**
**IL10RA**	**6.46**	**Lymphocyte infiltrate**
**SIT1**	**5.11**	**Lymphocyte infiltrate**
**TNFAIP8**	**4.25**	**Lymphocyte infiltrate**
**FYB**	**3.76**	**Lymphocyte infiltrate**
**PTPN7**	**3.68**	**Lymphocyte infiltrate**
**CCR5**	**3.38**	**Lymphocyte infiltrate**
**LILRB2**	**3.17**	**Lymphocyte infiltrate**
**PTPRC**	**3.13**	**Lymphocyte infiltrate**
**LY9**	**2.81**	**Lymphocyte infiltrate**
**SAMHD1**	**2.75**	**Lymphocyte infiltrate**
**CXCR6**	**2.59**	**Lymphocyte infiltrate**
**TAGAP**	**1.11**	**Lymphocyte infiltrate**
**TARP**	**1.10**	**Lymphocyte infiltrate**
CX3CR1	0.83	Lymphocyte infiltrate
TLR8	0.52	Lymphocyte infiltrate
**AIF1**	**205.19**	**Macrophage**
**CD68**	**95.04**	**Macrophage**
**FCGR3A**	**51.28**	**Macrophage**
**CD163**	**13.22**	**Macrophage**
ALOX15B	0.28	Macrophage
**CD33**	**4.77**	**Myeloid marker**
**CEACAM8**	**0.76**	**Myeloid marker**
**MPO**	**0.10**	**Myeloid marker**
**S100A9**	**33.70**	**Myeloid marker,MDSC**
**S100A8**	**3.62**	**Myeloid marker,MDSC**
FUT4	0.99	Myeloid marker,stem cell
**PYGL**	**103.74**	**Neutrophil**
**DGAT2**	**8.72**	**Neutrophil**
**KREMEN1**	**3.78**	**Neutrophil**
LRG1	0.95	Neutrophil
CA4	0.18	Neutrophil
**GNLY**	**14.35**	**NK activation**
**KLRB1**	**9.41**	**NK activation**
**KLRK1**	**6.28**	**NK activation**
**PRF1**	**4.35**	**NK activation**
**KLRG1**	**1.69**	**NK activation**
B3GAT1	0.35	NK activation
KLRF1	0.25	NK activation
FCGR3B	0.06	NK activation
NCR3	3.17	NK cell marker
**NFATC1**	**2.22**	**PD-1 signaling**
**PRDM1**	**0.48**	**PD-1 signaling**
**PTEN**	**10.70**	**PD-1 signaling,tumor marker**
**HIF1A**	**4.83**	**PD-1 signaling,tumor marker**

**PIK3CD**	**4.04**	**ᑭD-1 signaling,tumor marker**
**PTPN11**	**2.11**	**ᑭD-1 signaling,tumor marker**
**PIK3CA**	**1.80**	**ᑭD-1 signaling,tumor marker**
**FOXO1**	**1.64**	**ᑭD-1 signaling,tumor marker**
**MTOR**	**1.63**	**ᑭD-1 signaling,tumor marker**
**CDKN3**	**35.08**	**Proliferation**
**CCNB2**	**17.23**	**Proliferation**
**KIAA0101**	**14.69**	**Proliferation**
**CDK1**	**14.44**	**Proliferation**
**MAD2L1**	**13.34**	**Proliferation**
**FOXM1**	**7.70**	**Proliferation**
**TOP2A**	**3.96**	**Proliferation**
**BUB1**	**3.27**	**Proliferation**
**MELK**	**2.39**	**Proliferation**
**MKI67**	**2.06**	**Proliferation**
EGR2	0.54	T cell differentiation
LEXM	0.06	T cell differentiation
**NFKBIA**	**31.67**	**T cell receptor signaling**
**PTPN6**	**10.73**	**T cell receptor signaling**
**ZAP70**	**6.67**	**T cell receptor signaling**
**CBLB**	**1.55**	**T cell receptor signaling**
CD40LG	0.29	T cell receptor signaling
**M6PR**	**67.02**	**T cell regulation**
**ID3**	**36.04**	**T cell regulation**
**ID2**	**18.11**	**T cell regulation**
**IL18**	**14.42**	**T cell regulation**
**EBI3**	**6.09**	**T cell regulation**
**IL15**	**2.16**	**T cell regulation**
FOXP3	0.60	T cell regulation
**KLF2**	**11.11**	**T cell regulation,trafficking**
**PTPRCAP**	**98.36**	**TCR coexpression**
**CD3D**	**51.37**	**TCR coexpression**
**CD8B**	**14.82**	**TCR coexpression**
**CD3E**	**11.16**	**TCR coexpression**
**CD8A**	**7.47**	**TCR coexpression**
**LCK**	**7.40**	**TCR coexpression**
**CD247**	**6.47**	**TCR coexpression**
**CCR7**	**4.00**	**TCR coexpression**
**CD6**	**3.26**	**TCR coexpression**
**TIGIT**	**3.16**	**TCR coexpression**
**IL7R**	**2.46**	**TCR coexpression**
**GRAP2**	**2.20**	**TCR coexpression**
**CD3G**	**1.71**	**TCR coexpression**
**IL2RB**	**1.59**	**TCR coexpression**
**IKZF3**	**1.41**	**TCR coexpression**
**GPR18**	**1.36**	**TCR coexpression**
**LAMP3**	**1.15**	**TCR coexpression**
ITK	0.74	TCR coexpression
CRTAM	0.55	TCR coexpression
**MLANA**	**289.90**	**Tumor antigen**

CTAG2	0.43	Tumor antigen
MAGEA10	0.07	Tumor antigen
**RPS6**	**2155.35**	**Tumor marker**
**MYC**	**42.09**	**Tumor marker**
**MMP2**	**32.84**	**Tumor marker**
**AKT1**	**32.46**	**Tumor marker**
**PGF**	**22.19**	**Tumor marker**
**MMP9**	**19.04**	**Tumor marker**
**CDKN2A**	**17.50**	**Tumor marker**
**MAPK1**	**10.28**	**Tumor marker**
**EFNA4**	**7.65**	**Tumor marker**
**RB1**	**3.76**	**Tumor marker**
**PTK7**	**2.54**	**Tumor marker**
**TCF7**	**1.92**	**Tumor marker**
**KRT7**	**1.15**	**Tumor marker**
**NOTCH3**	**1.11**	**Tumor marker**
EGR3	0.56	Tumor marker
KRT5	0.50	Tumor marker
BRCA1	0.48	Tumor marker
IRS1	0.43	Tumor marker
TP63	0.03	Tumor marker
BRCA2	0.03	Tumor marker
EGFR	0.02	Tumor marker
PTGS2	0.02	Tumor marker
**SNAI2**	**6.28**	**Tumor marker,stemness**
**TWIST1**	**3.72**	**Tumor marker,stemness**
**SNAI1**	**2.03**	**Tumor marker,stemness**
**ZEB1**	**1.29**	**Tumor marker,stemness**
**IFITM1**	**380.71**	**Type I interferon signaling**
**IFITM2**	**343.11**	**Type I interferon signaling**
**IFI27**	**322.68**	**Type I interferon signaling**
**BST2**	**173.91**	**Type I interferon signaling**
**ISG15**	**139.67**	**Type I interferon signaling**
**IFIT3**	**21.98**	**Type I interferon signaling**
**ISG20**	**18.09**	**Type I interferon signaling**
**IFIT1**	**3.09**	**Type I interferon signaling**
**PSMB9**	**86.85**	**Type II interferon signaling**
**TAP1**	**73.35**	**Type II interferon signaling**
**IRF9**	**43.64**	**Type II interferon signaling**
**STAT1**	**38.74**	**Type II interferon signaling**
**ICAM1**	**37.34**	**Type II interferon signaling**
**CXCL10**	**23.69**	**Type II interferon signaling**
**CXCL9**	**16.14**	**Type II interferon signaling**
**IRF1**	**14.42**	**Type II interferon signaling**
**CYBB**	**10.50**	**Type II interferon signaling**
**GBP1**	**8.22**	**Type II interferon signaling**
**OAS1**	**7.24**	**Type II interferon signaling**
**CX3CL1**	**5.32**	**Type II interferon signaling**
**CXCL13**	**4.31**	**Type II interferon signaling**
**CIITA**	**2.84**	**Type II interferon signaling**

**BCL6**	**2.27**	**Type II interferon signaling**
**CXCL11**	**2.05**	**Type II interferon signaling**
**EIF2AK2**	**1.58**	**Type II interferon signaling**
FASLG	0.74	Type II interferon signaling
IL1B	0.71	Type II interferon signaling
CXCR5	0.39	Type II interferon signaling
TBX21	0.32	Type II interferon signaling
IFNG	0.26	Type II interferon signaling
