# Supplementary figures and images for: Changes in the TCRβ Repertoire and Tumor Immune Signature From a Cutaneous Melanoma Patient Immunized With the CSF-470 Vaccine: A Case Report
Source: Front Immunol. 2018 May 3;9:955. doi: 10.3389/fimmu.2018.00955 (PMC5944263; doi:10.3389/fimmu.2018.00955)

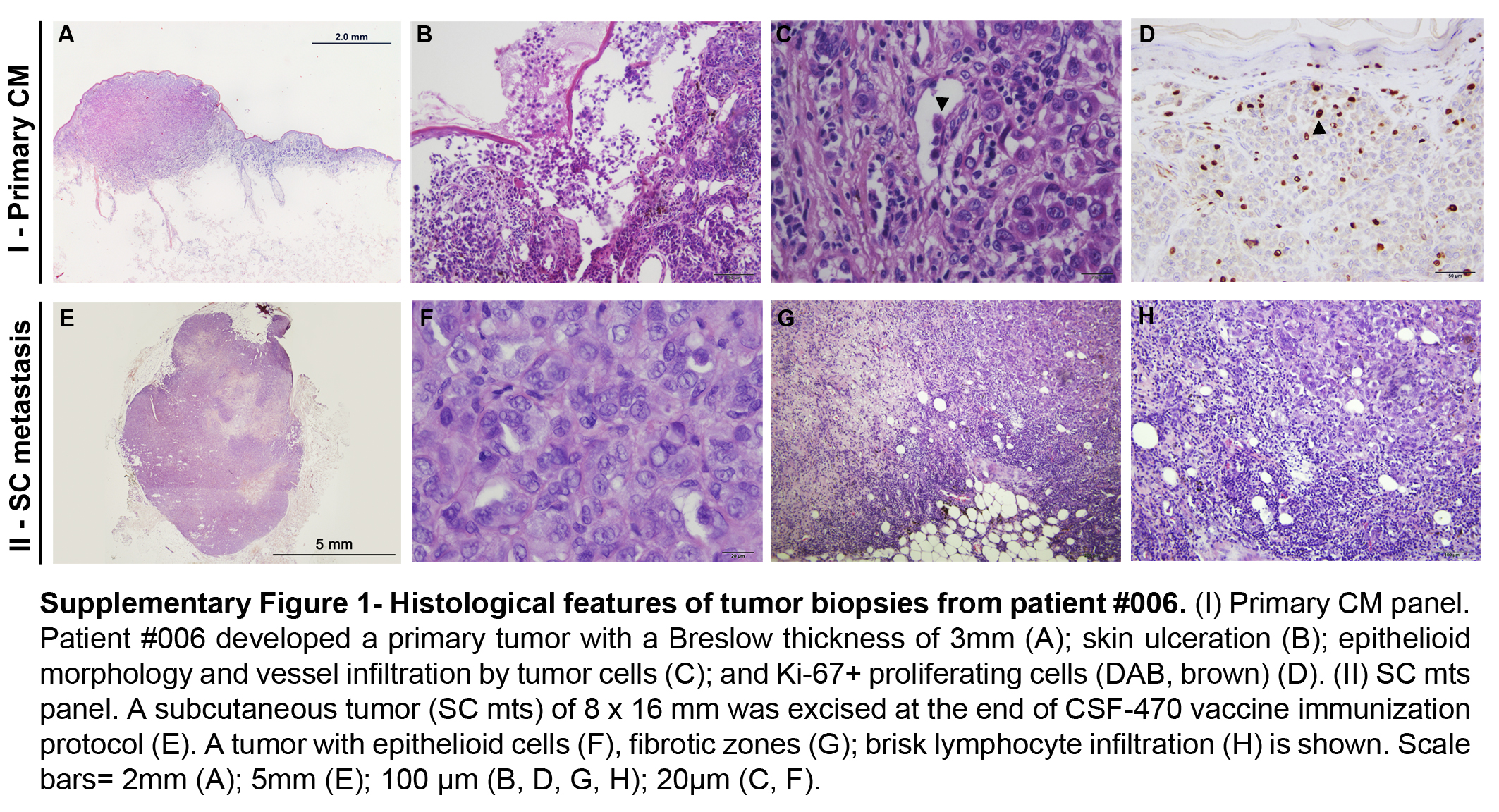

Supplement: Supplementary file 7 [file image_1.jpg]
