# Supplementary material for: Changes in the TCRβ Repertoire and Tumor Immune Signature From a Cutaneous Melanoma Patient Immunized With the CSF-470 Vaccine: A Case Report
Source: Front Immunol. 2018 May 3;9:955. doi: 10.3389/fimmu.2018.00955 (PMC5944263; doi:10.3389/fimmu.2018.00955)
Supplement: Supplementary file 8 [file image_2.PDF]

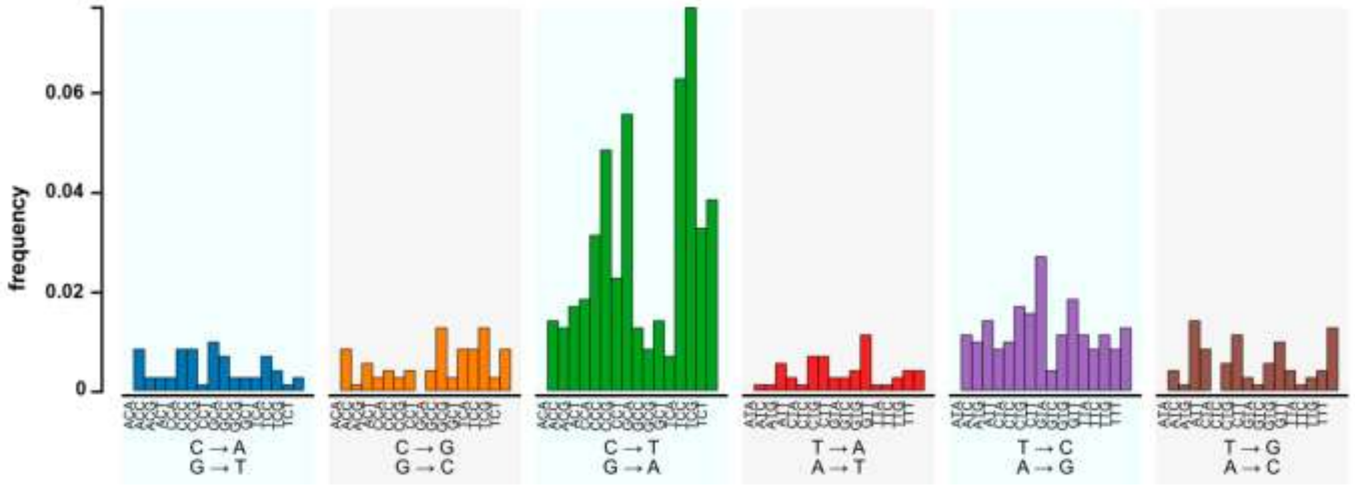

Supplementary Figure 2. Mutational signature of SC mts from patient #006. The profile of each signature is shown regarding the six substitution subtypes: C>A, C>G, C>T, T>A, T>C, and T>G.
