# Supplementary material for: Changes in the TCRβ Repertoire and Tumor Immune Signature From a Cutaneous Melanoma Patient Immunized With the CSF-470 Vaccine: A Case Report
Source: Front Immunol. 2018 May 3;9:955. doi: 10.3389/fimmu.2018.00955 (PMC5944263; doi:10.3389/fimmu.2018.00955)
Supplement: Supplementary file 9 [file image_3.PDF]

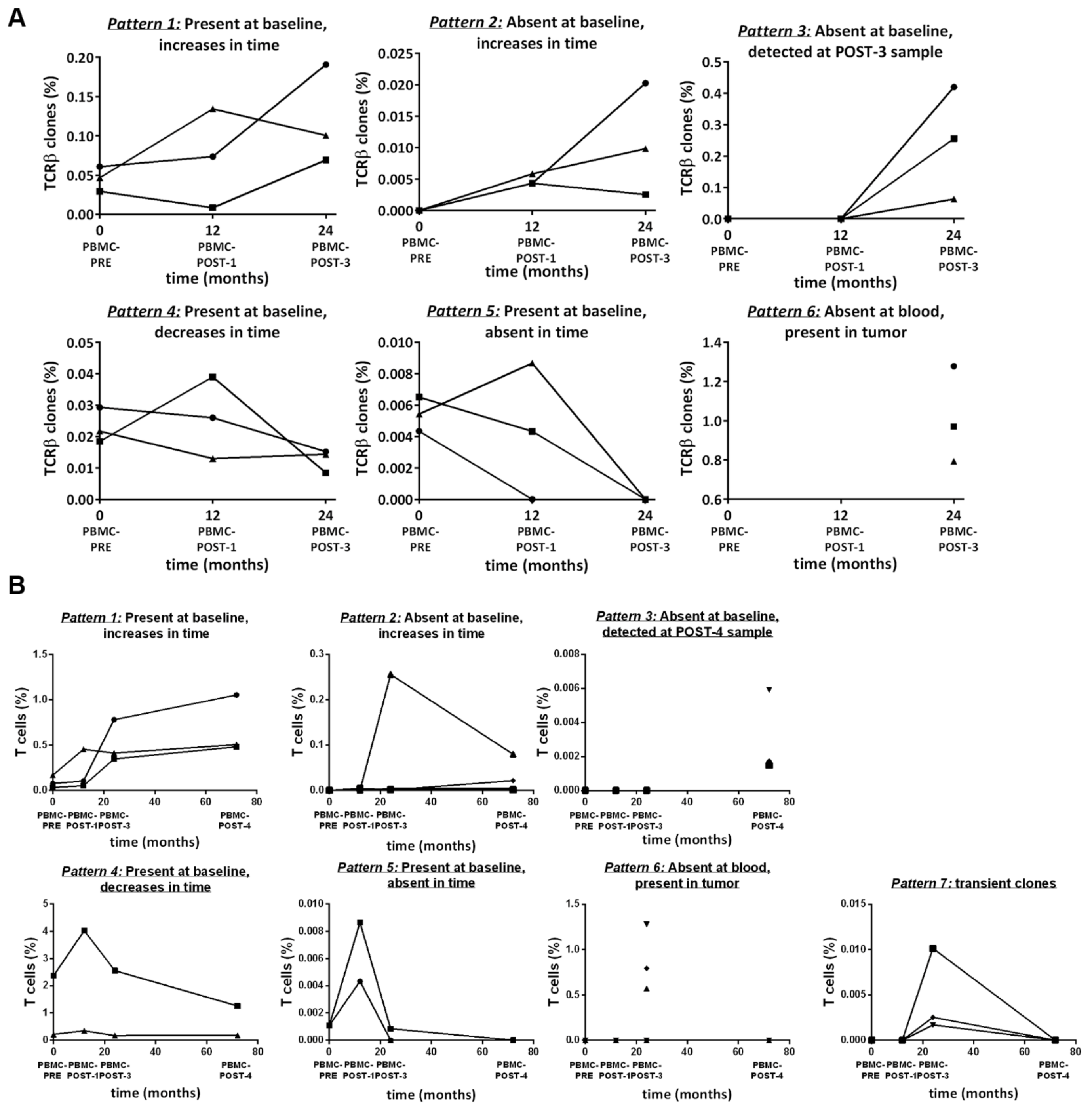

Supplementary Figure 3. Tracking of TCRβ clones from patient #006 throughout immunization with CSF-470 vaccine and follow-up. (A) Six patterns for TCRβ clone tracking were established for analysis in time at 0, 12 and 24 (PBMC-PRE, PBMC-POST-1 and PBMC-POST-3 samples). To illustrate each pattern, tracking of three different clones are shown (productive amino acid sequences). **(B)** Seven patterns for TCRβ clone tracking were established for analysis in time at 0, 12, 24 and 48 months (PBMC-PRE, PBMC-POST-1, PBMC-POST-3 and PBMC-POST-4 samples). Representative clones for each pattern are shown.
