# Supplementary material for: Changes in the TCRβ Repertoire and Tumor Immune Signature From a Cutaneous Melanoma Patient Immunized With the CSF-470 Vaccine: A Case Report
Source: Front Immunol. 2018 May 3;9:955. doi: 10.3389/fimmu.2018.00955 (PMC5944263; doi:10.3389/fimmu.2018.00955)
Supplement: Supplementary file 10 [file image_4.PDF]

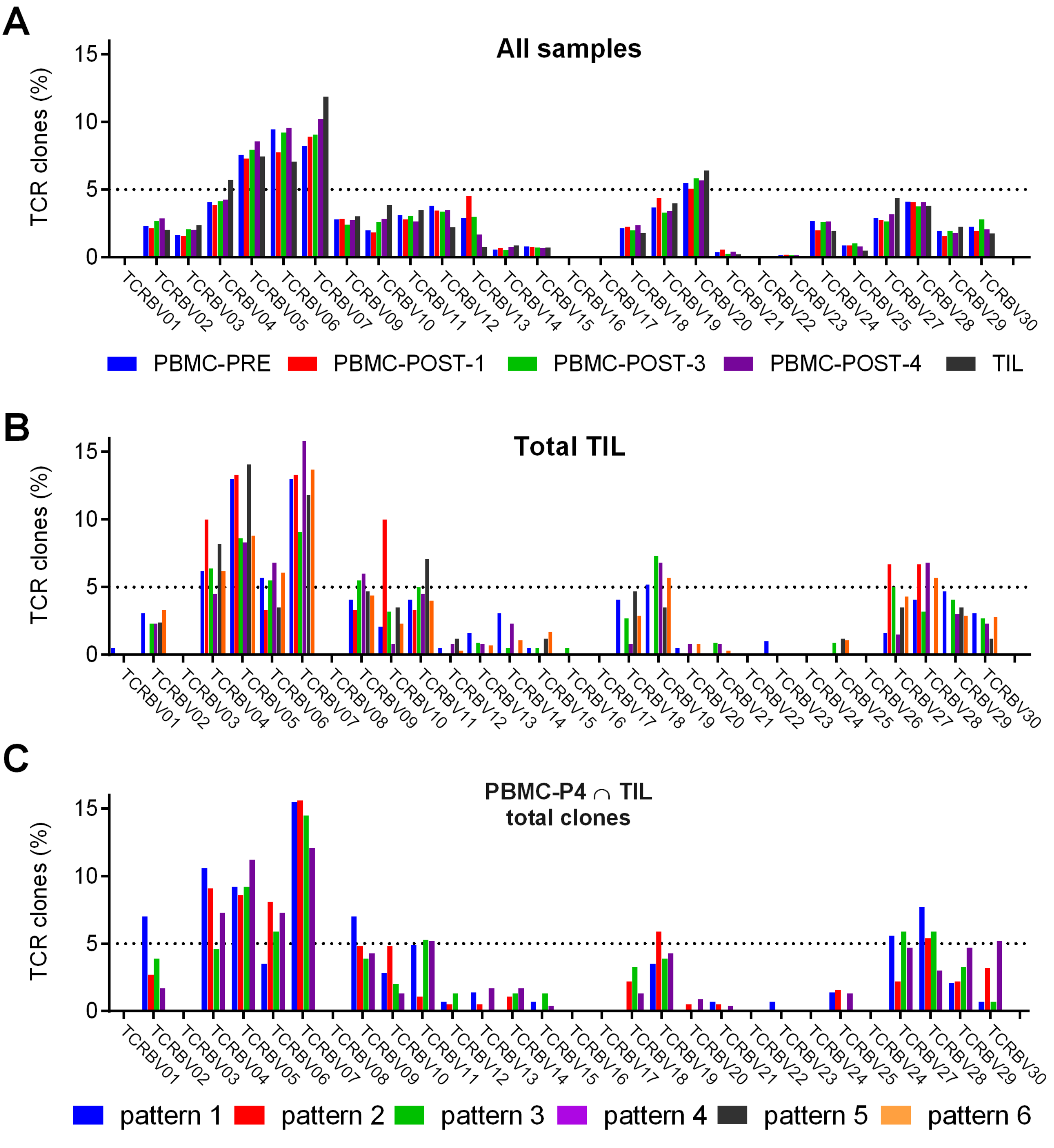

Supplementary Figure 4. Vβ gene usage of the TCRB (TCRβ) repertoire. (A) Distribution of Vß gene usage of T cell clones from all samples analysed (sum of productive frequencies). (B) Vß gene usage of TIL clones (n=1382) regarding their distribution patterns (frequency). (C) Vß gene usage of TIL clones (n=706) that persisted in the follow-up sample (PBMC-POST-4 ∩ TIL) regarding their distribution patterns (frequency). Samples: PBMC-PRE, PBMC-POST-1, PBMC-POST-3, PBMC-POST-4, TIL. *Patterns:* 1, present at baseline, increases in time; 2, absent at baseline, increases in time; 3, absent at baseline, detected at POST-4 sample; 4, present at baseline, decreases in time; 5, present at baseline, absent in time; 6, absent at blood, present in tumour.
