# Supplementary material for: Changes in the TCRβ Repertoire and Tumor Immune Signature From a Cutaneous Melanoma Patient Immunized With the CSF-470 Vaccine: A Case Report
Source: Front Immunol. 2018 May 3;9:955. doi: 10.3389/fimmu.2018.00955 (PMC5944263; doi:10.3389/fimmu.2018.00955)
Supplement: Supplementary file 11 [file data_sheet_1.PDF]

SUPPLEMENTARY MATERIAL

MATERIALS AND METHODS

PBMC samples, haplotype, and immune cell population analysis

During CASVAC-0401 protocol, peripheral blood samples were obtained from patient #6 at 0 (PRE), 6, 12, and 24 months (POST1-3) from immunization with CSF-470 vaccine (1); a follow-up sample was obtained at 48 months), 2-years after completing the protocol (POST-4). Peripheral blood mononuclear cells (PBMC) obtained by density gradient purification were incubated with anti-human MAbs: PerCP-CD3 (clone SK7), FITC-CD4 (clone RPA-T4), FITC-CD8 (clone RPA-T8), FITC-CD3 (clone UCHT1), APC-CD56 (clone B159) (BD Biosciences, San Jose, CA). For Tregs detection, $1x10^6$ PBMC were stained with mAbs (FITC-CD4, AF647- FoxP3 (clone 259D/C7) and PE-CD25 (clone M-A251) (BD Biosciences) following the manufacturer´s protocol. Lymphocytes were gated in FSC/SSC dot plot (≥30,000 events) and NK ($CD3^-CD56^+$), $CD4^+$, $CD8^+$ and Tregs ($CD4^+CD25^+FoxP3^+$) cells were determined. Isotype-matched irrelevant MAbs were used as negative controls. All samples were acquired on a BD FACSCalibur using Cellquest Pro software (BD Biosciences, Franklin Lakes, NJ, USA) and analyzed with FlowJo 7.6.2 software (FlowJo, Ashland, OR, USA).

HLA haplotype was determined from PBMC by Scisco Genetics (2); presenting haplotype HLA-A*11:01; -A*25:01; -B*51:01; -B*57:01; -C*06:02; -C*15:02; -DPA1*01:03; -DPB1*02:01; DPB1*04:01; -DQA1*:02:01; -DQA1*:03:01; -DQB1*03:02; -DQB1*03:03; -DRB1*04:01; DRB1*07:01; -DRB345*01:01; -DRB345*01:03.

Histopathological and immunohistochemical analysis of tumor biopsies

FFPE primary tumor and SC mts were studied (1). Histopathological features were determined according to AJCC-UICC staging (3). Proliferative index (PI) was determined by $Ki-67^+$ staining (4) (PI) (%): $Ki-67^+$tumor cells/($Ki-67^+$ tumor cells + $Ki-67^-$tumor cells)x100. PI was determined in 1 mm^2 tumor hot-spot zone (clone MIB-1, Dako). HLA-I expression (%) was determined in tumor tissues with clone EMR8-5 (Abcam). Tumor biopsies were stained with the following anti-human monoclonal Abs: CD8 (C8/144), CD20 (L26), CD45Ro (UCHL1) and CD68 (PG-M1) from Dako, Denmark; Foxp3 (236A/E7), CD11c (EP1347Y), GZMB (EPR8260) and PD-1 (NAT105) from Abcam, MA, USA; CD4 (1F6) from Novocastra, Wetzlar, Germany; and PD-L1 (E1L3N) from Cell-Signalling Technology, MA, USA. In situ Nick Translation (ISNT) was performed as previously described (5). The Avidin-Biotin-Peroxidase (ABC) system (Vectastain, Vector Labs) was used. Sections were examined by optical microscopy (Olympus BX40 microscope, DP2-BSW software) and digitalized pictures were analyzed with ImageJ software (NIH). Unless specified, total section counts were performed on whole slides and then different ratios were calculated.

Tumor Mutational Burden and Gene expression analysis

Total DNA and RNA from frozen SCmts were isolated with DNAzol and RNAzol respectively (Invitrogen); DNA extracted from PBMC was used as the germline reference. DNA and RNA were verified by spectrophotometry (2000 Nanodrop, ThermoFisher Scientific) and electrophoresis (2100 Agilent Bioanalyser, Agilent Genomics, Santa Clara, USA). Whole exon sequencing (WES) was performed in paired SC mts and blood DNA samples in an Illumina Hiseq platform (BGI, Hong Kong, China).

Total clean reads per sample were aligned to the human reference genome (GRCh38) using Burrows-Wheeler Aligner (BWA) (6). 97.71% mapped successfully. The mean sequencing depth on target regions were 65.41-fold. The average GC content was 45.40%. Substitution types statistics were obtained using SAMtools (6). To identify somatic single-nucleotide variants (SNVs) and insertions/deletions (InDels) present in the SC mts we used MuTect2 (7) from GATK version 3.8-0. Mutect2 was applied using Cosmic version 82 (8), dbSNP build 138 (9), removing soft clipped bases, and setting a TLOD threshold of 5.88. Identified variants were annotated with Variant Effect Predictor (10). Besides from MuTect2, manual curation for variants related to cancer driver genes (11) was performed, and 3 variants were added. Tumor mutational burden (TMB) was defined as the number of somatic, base substitution, and indel mutations per megabase of genome examined (12). Mutational signature of SC mts was determined with MutaGene (13). $BRAF^{V600}$ status was determined after DNA extraction from tumor biopsies, PCR amplification and Sanger sequencing as previously described (1).

For RNA-seq total RNA was treated with DNase I, and Oligo (dT) are used to isolate mRNA. After fragmentation, cDNA was synthesized using the mRNA fragments as templates. Short fragments were purified, end-repared and single nucleotide A (adenine) addition. After that, the short fragments were connected with adapters. The suitable fragments were selected for the PCR amplification. During the QC steps, Agilent 2100 Bioanaylzer and ABI StepOnePlus Real-Time PCR System were used in quantification and qualification of the sample library. Then the library was sequenced using Illumina HiSeq 4000 platform Illumina, with more than 20 M high-quality single-end reads (BGI, Hong Kong, China). Quality control of reads was performed with FASTX-Toolkit (14) and FastQC (15). Reads were aligned to the latest human Hg38 reference genome using the STAR spliced read aligner (16). Fragment counts were derived using HTSeq package (17). Transcript abundance is shown as fragments per kilobase of exon per million reads mapped (FPKM). RNA-Seq were manually curated to search for the expression of transcripts that are related to the immune function (18) and of several melanoma associated Ags, selected that have been previously shown to be immunogenic in CM pts (19). RNASeq data from the SC mts was uploaded to the European Nucleotide Archive (ENA, EMBL EBI); the corresponding accession number is PRJEB23421, ENA).

TCRβ immune repertoire analysis

Genomic DNA was isolated from total PBMC samples (PRE-PBMC, POST-1-PBMC, POST-3-PBMC, POST-4-PBMC) FFPE SC mts and using RecoverAll (Ambion) and DNAzol (Invitrogen); DNA was verified by spectrophotometry (Nanodrop). High-

throughput next-generation sequencing of the T-cell receptor beta (TCRβ) CDR3 region was performed at survey resolution and analysed through Immunoseq platform (Adaptive Biotechnologies, USA). POST-4-PBMC sample TCRβ sequencing was performed at deep resolution.

The frequency of each TCRβ clone was calculated by dividing the number of reads per unique TCRβ sequence by an overall number of TCRβ sequence reads per sample. To perform analysis of the nucleotide and amino acid sequences from TCRβ clones, only productive rearrangements were considered, in-frame unique sequences without stop codons. Unique nucleotide/aminoacid productive rearrangements (TCRβ clones) for each sample were: PBMC-PRE (60053/58491), PBMC-POST-1 (14681/14455), PBMC-POST-3 (65248/63852,), PBMC-POST-4 (209103/196336), TIL (1411/1382) **(Supplementary tables 2-6)**.

TCRβ immune repertoire distribution: TOP100 are defined as the 100 most-frequent clones, ordered by frequency. Top-quartile clones (TQ) or TOP-25 are the number of cumulative clones, ordered by frequency, that make up the 25% most-frequent TCRβ clones; while TOP-50 is the number of cumulative clones that make up the 50% most-frequent TCRβ clones.

Blood TCRβ clone-tracking patterns throughout CSF-470 immunization (Figure 5): 1, present at baseline, increases in time (P0<P1<P3, P0>0; P0>P1<P3, P3>P0, P0>0; P0 <P1>P3, P3>P1, P0>0); 2, absent at baseline, increases in time (P0=0; P1>0; P1<P3; P0=0; P1>0; P1>P3); 3, absent at baseline, detected at POST-3 sample (P3>0; P1=P0=0); 4, present at baseline, decreases in time (P0>P1>P3, P3>0; P0<P1>P3; P3<P0, P3>0; P0>P1< P3, P3<P0); 5, present at baseline, absent in time (P0 >0; P1=P3=0; P0 >P1, P1>0, P3=0; P0<P1, P0>0, P3=0); 6, absent at blood, present in tumor (TIL>0; P3=P1=P0=0).

Blood TCRβ clone-tracking patterns throughout CSF-470 immunization and follow-up (Supplementary Figure 3): 1, present at baseline, increases in time (P0>0;P4>P0); 2, absent at baseline, increases in time (P0=0;P1<P3<P4; P0=0;P1>P3<P4; P0=0;P1>P3>P4;P4>0; P0=0;P1<P3>P4;P4>0); 3, absent at baseline, detected at POST-4 sample (P0=P1=P3=0;P4>0); 4, present at baseline, decreases in time (P0>0;P4<P0;P4>0); 5, present at baseline, absent in time (P0>0;P4=0); 6, absent at blood, present in tumor (P0=P1=P3=P4=0;FFPE>0); 7, transient clones (P0=0;P1>P3;P4=0; P0=0;P1<P3;P4=0).

REFERENCES

1. Alexandrov LB, Nik-Zainal S, Wedge DC, Aparicio SAJR, Behjati S, Biankin A V., Bignell GR, Bolli N, Borg A, Børresen-Dale A-L, et al. Signatures of mutational processes in human cancer. *Nature* (2013) **500**:415–421. doi:10.1038/nature12477
2. Nelson WC, Pyo CW, Vogan D, Wang R, Pyon YS, Hennessey C, Smith A, Pereira S, Ishitani A, Geraghty DE. An integrated genotyping approach for HLA and other complex genetic systems. *Hum Immunol* (2015) **76**:928–938.

doi:10.1016/j.humimm.2015.05.001

3. Balch CM, Gershenwald JE, Soong SJ, Thompson JF, Atkins MB, Byrd DR, Buzaid AC, Cochran AJ, Coit DG, Ding S, et al. Final version of 2009 AJCC melanoma staging and classification. *J Clin Oncol* (2009) **27**:6199–6206. doi:10.1200/JCO.2009.23.4799

4. Ladstein RG, Bachmann IM, Straume O, Akslen LA. Ki-67 expression is superior to mitotic count and novel proliferation markers PHH3, MCM4 and mitosin as a prognostic factor in thick cutaneous melanoma. *BMC Cancer* (2010) **10**:140. doi:10.1186/1471-2407-10-140

5. Zubieta MR, Furman D, Barrio M, Bravo AI, Domenichini E, Mordoh J. Galectin-3 expression correlates with apoptosis of tumor-associated lymphocytes in human melanoma biopsies. *Am J Pathol* (2006) **168**:1666–75. doi:10.2353/ajpath.2006.050971

6. Li H, Durbin R. Fast and accurate long-read alignment with Burrows–Wheeler transform. *Bioinformatics* (2010) **26**:589–595. doi:10.1093/bioinformatics/btp698

7. Cibulskis K, Lawrence MS, Carter SL, Sivachenko A, Jaffe D, Sougnez C, Gabriel S, Meyerson M, Lander ES, Getz G. Sensitive detection of somatic point mutations in impure and heterogeneous cancer samples. *Nat Biotechnol* (2013) **31**:213–9. doi:10.1038/nbt.2514

8. Forbes SA, Beare D, Boutselakis H, Bamford S, Bindal N, Tate J, Cole CG, Ward S, Dawson E, Ponting L, et al. COSMIC: somatic cancer genetics at high-resolution. *Nucleic Acids Res* (2017) **45**:D777–D783. doi:10.1093/nar/gkw1121

9. Sherry ST, Ward MH, Kholodov M, Baker J, Phan L, Smigielski EM, Sirotkin K. dbSNP: the NCBI database of genetic variation. *Nucleic Acids Res* (2001) **29**:308–11.

10. McLaren W, Gil L, Hunt SE, Riat HS, Ritchie GRS, Thormann A, Flicek P, Cunningham F. The Ensembl Variant Effect Predictor. *Genome Biol* (2016) **17**:122. doi:10.1186/s13059-016-0974-4

11. Vogelstein B, Papadopoulos N, Velculescu VE, Zhou S, Diaz LA, Kinzler KW. Cancer Genome Landscapes. *Science (80-)* (2013) **339**:1546–1558. doi:10.1126/science.1235122

12. Chalmers ZR, Connelly CF, Fabrizio D, Gay L, Ali SM, Ennis R, Schrock A, Campbell B, Shlien A, Chmielecki J, et al. Analysis of 100,000 human cancer genomes reveals the landscape of tumor mutational burden. *Genome Med* (2017) **9**:34. doi:10.1186/s13073-017-0424-2

13. Goncearenco A, Rager SL, Li M, Sang Q-X, Rogozin IB, Panchenko AR. Exploring background mutational processes to decipher cancer genetic heterogeneity. *Nucleic Acids Res* (2017) **45**:W514–W522. doi:10.1093/nar/gkx367

14. FastX. Available at: http://hannonlab.cshl.edu/fastx_toolkit/

15. Fastqc. Available at: http://www.bioinformatics.babraham.ac.uk/projects/fastqc/

16. Dobin A, Davis CA, Schlesinger F, Drenkow J, Zaleski C, Jha S, Batut P, Chaisson M, Gingeras TR. STAR: ultrafast universal RNA-seq aligner. *Bioinformatics* (2013)

29:15–21. doi:10.1093/bioinformatics/bts635

17. Anders S, Pyl PT, Huber W. HTSeq--a Python framework to work with high-throughput sequencing data. *Bioinformatics* (2015) **31**:166–9. doi:10.1093/bioinformatics/btu638

18. Paluch BE, Glenn ST, Conroy JM, Papanicolau-Sengos A, Bshara W, Omilian AR, Brese E, Nesline M, Burgher B, Andreas J, et al. Robust detection of immune transcripts in FFPE samples using targeted RNA sequencing. *Oncotarget* (2017) **8**:3197–3205. doi:10.18632/oncotarget.13691

19. Bassani-Sternberg M, Bräunlein E, Klar R, Engleitner T, Sinitcyn P, Audehm S, Straub M, Weber J, Slotta-Huspenina J, Specht K, et al. Direct identification of clinically relevant neoepitopes presented on native human melanoma tissue by mass spectrometry. *Nat Commun* (2016) **7**:13404. doi:10.1038/ncomms13404
